# Credibility dilemmas under the Paris agreement: explaining fossil fuel subsidy reform references in INDCs

**DOI:** 10.1007/s10784-022-09581-8

**Published:** 2022-06-22

**Authors:** Christian Elliott, Steven Bernstein, Matthew Hoffmann

**Affiliations:** Department of Political Science, University of Toronto, Toronto, Canada

**Keywords:** Climate change, International norms, Fossil fuel subsidies, NDCs, Paris agreement, Environmental politics

## Abstract

Fossil fuel subsidies are a market distortion commonly identified as an obstacle to decarbonization. Yet  due to trenchant political economic risks, reform attempts can be fraught for governments. Despite these concerns, an institutionally and economically diverse group of states included references to fossil fuel subsidy reform (FFSR) in their Intended Nationally Determined Contributions (INDCs) under the Paris Agreement. What conditions might explain why some states reference politically risky reforms within treaty commitments, while most others would not? We argue that the Article 4 process under the Paris Agreement creates a “credibility dilemma” for states–articulating ambitious emissions reduction targets while also defining national climate plans engenders a need to seek out appropriate policy ideas that can justify overarching goals to international audiences. Insomuch as particular norms are institutionalized and made salient in international politics, a window of opportunity is opened: issue advocates can “activate” norms by demonstrating how related policies can make commitments credible. Using mixed methods, we find support for this argument. We identify contextual factors advancing FFSR in the lead-up to the Paris Agreement, including norm institutionalization in regimes and international organization programs as well as salience-boosting climate diplomacy. Further, we find correspondences between countries targeted by transnational policy advocates and FFSR references in INDCs, building on the momentum in international politics more generally. Though drafting INDCs and NDCs is a government-owned process, the results suggest that understanding their content requires examining international norms alongside domestic circumstances.

## Introduction

New Net Zero commitments and ambitious climate plans are now ubiquitous. Yet, sometimes in the same jurisdictions making bold announcements, fiscal policies that subsidize the consumption or production of fossil fuels often remain stubbornly locked in. The Organization for Economic Cooperation and Development (OECD) estimates that, among its member and partner economies, the aggregate estimated volume of price supporting subsidies was between USD 150 Billion to USD 250 Billion annually between 2010 and 2016 (OECD, 2018). Even at the low end, it outstrips the estimated total revenue raised from all forms of carbon pricing, globally, in the same period (The World Bank, Ecofys, & Vivid Economics, 2016).

Arguably, the same distributional conflicts that undermine or water down climate policies (see Aklin & Mildenberger, 2020; Colgan et al., 2021) drive the persistence of fossil fuel subsidies. Convergent pressures from powerful business interests and citizens resistant to higher fuel costs raise the political stakes for leaders considering reform. On the one hand, eliminating subsidies and reclaiming fiscal slack is advantageous to the state. On the other, as Inchauste and Victor describe, the political economic challenges make fossil fuel subsidy reform (FFSR) “a task nearly always fraught with large political costs and risks” (2017, p. 13).

Nonetheless, 15 countries made references to FFSR in their Intended Nationally Determined Contributions (INDCs) under the Paris Agreement. If we accept the that INDCs/NDCs map state interests (Keohane & Victor, 2016) and that the transparency of progress reporting under Paris invites naming and shaming that can sanction policy reversals (Falkner, 2016; Weikmans et al., 2020), we would seldom expect states to signal interest in high-risk policies voluntarily, relative to easier alternatives. Further, given insights from the political economy literature, we might expect clear patterns of national variation with regards to references, whether as a function of levels of development or fossil fuel production. Yet countries like Iran, China, and Ghana included references alike. Under what conditions would these states, despite no clear economic or institutional commonality, decide to reference a contentious issue like FFSR under an international environmental agreement?

Answering this question in the context of the Paris Agreement is important for at least three reasons. First, we should be concerned with the content of NDCs^1^ because commitments vary considerably by country, unlike in other international environmental agreements. NDCs often involve general or nonspecific language to justify emissions reductions, so attention to these justifications and whether they entail new commitments is important. Second, though only fifteen countries made FFSR references, the group (including China and India) are responsible for over 40% of global CO_2_ emissions from fossil fuel consumption based on the International Energy Agency’s 2018 estimates (IEA, 2020), and includes some of the largest providers of consumer-facing energy and petroleum subsidies in the world (Shirai & Adam, 2017). The practical importance of studying this group is therefore significant. Finally, if bottom-up voluntary commitments become an increasingly common feature of international environmental agreements, understanding the underlying dynamics determining their substance is essential for scholarship.

In addressing the research question, we first assert that what countries include in their NDCs can be understood as a response to a credibility dilemma: where states are encouraged to take ambitious action, the exact policies and processes to achieve their commitments are left open-ended and “nationally” determined. States have to substantiate their ambition levels credibly to their peers, populating NDCs with actions that conform to a “logic of appropriateness,” adhering to a “structure of rules and conceptions of identities” (March & Olsen, 1996, p. 250). In other words, they will want to appear to be following expectations identified by general treaty commitments, in part through referencing policies seen as legitimate or appropriate.

To explain specific policy references like FFSR, we make a two-step argument. First, the degree to which a particular policy is considered appropriate is a function of its support by international norms and institutions more broadly. This assertion is well established in the literature on legitimacy in global governance where extant norms and institutions often define appropriate policies and actions (Barnett & Coleman, 2005, p. 598; Bernstein, 2001, 2011; Weber, 1994, p. 7). Second, a norm can be “activated” by transnational policy actors who make such policies salient for states seeking to substantiate their commitments. The credibility dilemma creates a window of opportunity for issue advocates advancing policies linked to even relatively weakly institutionalized norms, as states search for practical (and appropriate) actions to justify their climate goals.

Using mixed methods, we find evidence to support this argument. First, we examine INDC references and find that FFSR is wielded as a justification to support the credibility or ambition of commitments. Second, we document the institutionalization of FFSR in international agendas and international organizations (IOs), coinciding with issue-specific climate diplomacy which energized attention during and prior to The Conference of Parties (COP) 21. Third, and building on these conditions, we find that countries engaged by issue advocates were more likely to reference FFSR in their INDCs, even after considering differences in institutional and economic country characteristics. Such characteristics did not appear to moderate the “success” of advocacy, either. Though political economic constraints, i.e., material or institutional factors that structure interests, still matter in explaining what we observe (especially given the absence of strong commitments to FFSR in INDCs), the results suggest that NDCs are not merely expressions of domestic policy priorities but are also co-constitutive with evolving norms in international society. While domestic processes of issue normalization or policy experimentation are likely also an essential part of the story, a focus on international norms is an important rejoinder for understanding how justifications for climate action are constituted.

The remainder of the paper is organized as follows. First, we review the literature on the political economy of fossil fuel subsidies and their reform. Second, we consider the Paris Agreement’s structure for NDCs and outline our argument. Third, we describe our analytical strategy. Fourth, we unpack the content of FFSR references and, using a mixed methods approach, examine the role of contextual conditions shaping the salience and appropriateness of FFSR, and analyze patterns of issue advocacy by transnational policy actors in the lead up to the Paris Agreement. Finally, we summarize and discuss our findings and suggest areas for further research.

## The FFSR challenge

Fossil fuel subsidies, provided by states to decrease the cost of consuming or producing fossil fuel-based energy, are an often overlooked yet critical dimension of the transition to a low-carbon economy. Subsidies can be focused on consumers by controlling fuel and energy prices to reduce exposure to fluctuating international petroleum costs and to increase energy access. Or, subsidies can target energy producers, driven by concerns around economic competitiveness among fossil fuel exporting states (Beaton & Lontoh, 2010; Fattouh & El-Katiri, 2013; Victor, 2009). In either case, fossil fuel subsidies become difficult to reverse once established. With consumer subsidies, the political difficulty of reform has been made clear in instances like Nigeria in 2012: attempts to remove gasoline and diesel subsidies resulted in two weeks of civil unrest and nearly immediate policy reversal (Peralta, 2012). The case of Canada is illustrative of the challenge of reversing subsidies on the production side. Despite progress in the early 2010s in eliminating millions of dollars of fossil fuel subsidies in the federal budget (Green Budget Coalition, 2013), subsidies continue to expand in new and obvious ways (e.g., subsidizing new Liquified Natural Gas infrastructure) as well as in more subtle and difficult to measure ways (e.g., liability protections, overpayment for pipeline assets, etc.) (Corkal et al., 2020), undoubtedly due to the persistent political influence of fossil fuel companies.

A growing inter-disciplinary literature has attempted to evaluate how that might change, especially with regards to consumer subsidies, analyzing instances of reform attempts or making use of subsidy estimates from organizations like the International Energy Agency (IEA). Among scholars, there is some disagreement about the relative weight of economic and institutional factors in increasing the likelihood of reform. Cheon et al. (2013), for example, find that authoritarianism, poor institutional capacity, and Organization of the Petroleum Exporting Countries (OPEC) membership are significant correlates for higher gasoline subsidies in their longitudinal analysis. Such conclusions align with case studies that evaluate the likelihood of reform as a function of the size of petroleum rents, the degree of policy transparency, and the power of special interests within countries (Inchauste & Victor, 2017; Koplow, 2014; Lockwood, 2015). Conversely, recent work modeling variation in gasoline prices between 2003 and 2015 concluded that government indebtedness, national income, and fossil fuel wealth were most critical to variance in taxes or subsidies; institutional characteristics like governance effectiveness were not significant (Mahdavi, Martinez-Alvarez, and Ross 2020; Ross et al., 2017). Instead, the authors suggest that successful reforms involved non-systematic political factors creating contingent moments of opportunity. Outside of the institutional-economic axis, research has also examined policy design. Whether the population has been adequately informed about price changes, whether subsidy removal is complemented with conditional cash transfers or other welfare substitutes, and how energy price volatility is managed otherwise differentiates successful efforts from failed ones (Inchauste & Victor, 2017; Rentschler & Bazilian, 2017).

Other scholars have examined FFSR as an international norm, especially since the United States chose subsidy reform as a G20 priority in 2009, tasking the IEA, OPEC, OECD, and the World Bank with informing and facilitating a country peer-review fossil fuel subsidy stocktaking (Van de Graaf & Blondeel, 2018). Downstream of this critical juncture, studies have examined how international normalization has affected domestic policymaking. Issue-focused non-governmental organizations (NGOs) like the Global Subsidies Initiative (GSI)^2^ took on catalytic roles and supported reform efforts in a number of states including Indonesia and India (Lemphers, Bernstein, & Hoffmann, 2018). Additionally, through technical assistance and conditionalities in loans, the World Bank and the International Monetary Fund (IMF) have supported reform in countries like Egypt (Skovgaard & van Asselt, 2019). Taken together, these initiatives, policies, and general support suggest the growing institutionalization of FFSR as a norm among major economies, leading international economic institutions, and transnational stakeholders. Thus, the social structural conditions that define reform as an appropriate action has become increasingly present.

However, the activation of reform policies has been weak. Estimates by the Overseas Development Institute and Oil Change International demonstrate that G20 country commitments, in their first five years, accomplished little (Bast, Doukas, Pickard, van de Burg, & Whitley, 2015), though Smith and Urpelainen (2017) argue that these commitments nonetheless increase reputational costs associated with policy reversal. While there is certainly evidence of advocacy manifesting change in some contexts, the degree to which it can overcome the political economic challenges of FFSR is less clear (van Asselt et al., 2018).

Moreover, FFSR-related agreements are few and far between. Compared to subsidies for fisheries or renewable energy that are routinely challenged in the World Trade Organization, fossil fuels have received little attention (De Bièvre et al., 2017; Verkuijl et al., 2019). Multilateral efforts in the G20 and APEC similarly entail relatively vague and unformalized commitments. On the other hand, the 2015 Paris Agreement, with Article 4 requiring each party to prepare individual plans for greenhouse gas (GHG) emissions reductions, involves references to FFSR that have remained unexamined in the academic literature. This paper addresses this gap by exploring the drivers and implications of these references, breaking ground on how formal international environmental agreements shape and are shaped by the politics of fossil fuel subsidy reform.

## Explaining FFSR in INDCs

Under Article 4 of the Paris Agreement, each party is required to outline and transparently report on GHG emissions reduction targets, means of reductions, and progress made (UNFCCC, 2015). Starting with the “Intended Nationally Determined Contributions” (INDCs)^3^ that were prepared in the lead-up to COP 21, commitments are intended to be updated and ratcheted up every five years. Currently, most theories discussing state commitments to formal international environmental agreements rely on the premise of collective commitment to shared and singular rules: treaties, protocols, and conventions that involve standardized, if often differentiated, responsibilities. The Paris Agreement is relatively unique in having a common and legally binding core goal combined with independently formulated national plans on how that goal is to be achieved.

The flexibility of NDCs arguably reveals state preferences with regard to climate change abatement (see Keohane & Victor, 2016). Certainly, the failure of the first round of submissions (2015–2020) to collectively orient countries to a trajectory of 2 degrees Celsius of warming (UNEP, 2016), despite that being an explicit objective of the Paris Agreement,^4^ is a potential indicator of their aggregate cautiousness and their reliability as a means for interpreting what states are willing to do. Further, the requirements of Article 4, the enhanced transparency framework in Article 13, and the global stocktaking in Article 14 should induce reputational and political costs to “cheap talk” (high stated ambitions and poor performance) in the face of domestic and international audiences (Falkner, 2016; Keohane & Oppenheimer, 2016). Though Paris may have less in the way of formal accountability mechanisms in comparison to other international treaties, an active global civil society of scientists, climate activists, and NGOs could act as a strong supplement (Campbell-Duruflé, 2021; Karlsson-Vinkhuyzen et al., 2018; van Asselt, 2016; Weikmans et al., 2020).

These background conditions sketch the outline of our core empirical puzzle: if NDCs represent strategic choices motivated by state interests, and also involve risks and rewards in light of international audiences, one would expect that a thorny issue like FFSR would be substituted for a safer bet and likely ignored altogether. Yet some states, including several major emitters, include references to FFSR.

To make sense of this puzzle, we first interrogate what political work an NDC does. We can broadly categorize two parallel political purposes or motivating logics. The first is compliance-based: in order to adhere to the Articles of the Agreement, developed countries define emissions reduction targets pegged to baseline years with corresponding timelines for meeting stated objectives and developing countries define mitigation actions and work towards economy-wide mitigation goals. These commitments are modified by both state preferences for abating climate change and international legal principles (common but differentiated responsibilities and respective capabilities). A second political purpose of NDCs, we argue, is to force a response to a credibility dilemma generated by the demands for greater ambition (e.g., Article 2, Article 4.3, Article 4.5) and the simultaneous requirement for each country to justify exactly how that ambition could be actualized (e.g.., Article 4.2).

Solving the credibility dilemma entails making commitments believable to an international audience by referencing policies and issue positions that justify the achievability of climate goals and ambition levels. But where do these references come from? As security studies scholars will highlight, a country’s past actions will be an important and obvious source of credibility for future commitments. At the same time, not all extant policy programs are feasible to scale up and some may no longer be considered a credible means of achieving future goals. Further, the importance of ambition makes the past, by definition, insufficient: states have to exceed prior trajectories of GHG mitigation in order to limit warming to 2 degrees Celsius. Inevitably, new approaches and positions will be considered. In either case and especially when political processes generate ambiguity and uncertainty, the choice of which policies to reference depends on an intersubjective evaluation of *what counts* as an appropriate policy position, i.e., its credibility as a solution given the circumstances of the country in question and its recognition as a viable and appropriate response by authoritative international actors and institutions. In other words, states want to be credible and secure a reputation as a committed actor in addressing climate change, but they may have to search for which practical actions will demonstrate credibility. The more a country justifies commitments on the back of appropriate policy choices or issue stances, the more credible their commitments stand to appear to other actors. If a commitment requires justification given external audiences, then policy references would not merely be the consequence of aggregated domestic preferences, though such interests surely influence boundaries of possibility.

From a constructivist standpoint, the appropriateness of a policy (and the ideas they embody) is relational and dynamic. The international system, as an evolving social structure, helps define appropriate action for states through reification or advancement of particular norms. Norms, as roadmaps for action, advance recursively as advocates succeed in catalyzing institutionalization in international agreements and regimes, as well as through domestication in national policies (Finnemore & Sikkink, 1998). International norms define and redefine which actions are considered appropriate and legitimate, or not, for states given their particular roles and circumstances (Finnemore, 1996). This dynamic persists even as norms are adapted and redefined to align with cognitive and normative priors as they become “localized” or enacted in particular countries (Acharya, 2004; Coe, 2019). Norms may be formalized in international agreements or law but can also be institutionalized in policies and programs of major international institutions, international standards, declarations and statements of world leaders and governments, and through wide acceptance within global civil society.

While extant norms provide a basis for credibility, the Paris Agreement itself is only vaguely suggestive of specific policy actions. Further, as prior research has highlighted, FFSR has not become so normalized that its appropriateness is entirely taken for granted. Indeed, political economic factors may create or support domestic interests that would line up against such a norm. The credibility dilemma of NDCs and the nascent institutionalization of a FFSR norm may be insufficient for generating references among countries searching for practical solutions to emissions reductions. Yet, their concurrance may open a window of opportunity for international and transnational actors to advocate for FFSR as a credible policy choice. It is only when norms become completely taken for granted that we might expect them to shape action without persuasion. When advocates make particular policies (like subsidy removal) salient as a credible solution to particular problems (GHG emissions) for a given country, they “activate” emerging norms in international society (that fossil fuel subsidies are inefficient and environmentally problematic). In this sense, norm activation^5^ describes instances where latent norms that *could* prescribe appropriate action become concrete in satisfying a country’s needs for legitimacy and credibility in political processes. This two-step argument (institutionalization and activation) provides the basis for explaining the variation that we observe.

More concretely, we would first expect to see FFSR institutionalized in ways that build momentum and salience as it becomes intersubjectively understood as a legitimate and appropriate policy response to climate change, especially in the lead-up to the Paris Agreement. Second, we would anticipate a higher degree of referencing FFSR in INDCs among countries engaged by transnational policy actors advocating for FFSR. In the subsequent sections, we draw on diverse evidence to assess these hypotheses. 

## Analytical approach

We empirically evaluate our argument in three stages. First, we unpack the references themselves to qualitatively interrogate how FFSR was wielded in the context of INDCs. Then, we turn to the various conditions exerting themselves in the lead-up to the Paris Agreement in order to understand the degree to which subsidy reform was made salient and available as a policy consideration more broadly. Finally, we use data from 2015 to examine patterns across countries with and without FFSR references, analyzing the extent to which references were associated with targeted issue advocacy by transnational and international policy actors.

Across all stages, we rely on interviews as well as triangulating evidence from primary documents and secondary sources. The third stage relies on both quantitative and qualitative evidence. We conducted 15 semi-structured interviews with issue experts across sectors (see Appendix 1), selecting interviewees based on their expertise and knowledge with regard to the Paris Agreement and FFSR. Diversity of organizational representation was prioritized. In many cases, we also used snowball sampling to identify key respondents. Among the final relevant sampling frame (37 individuals), the response rate was approximately 40%. Non-responses were correlated to individuals in current positions within government. No other patterns of non-response were identified. Interviews focused on three main topics: drivers, mechanisms, and implications of FFSR and INDCs. Interview guides and questions addressed which aspects interviewees had the most expertise on, as well as to fit the work experience of the individual. We reviewed and summarized coded interviews to identify maximally supported conclusions. Where possible, we validated information from interviews by checking additional sources.

## Results

### Fossil fuel subsidy reform references

Part of the puzzle of FFSR references is the apparent heterogeneity of the countries who made them. Replicating Terton et al. (2015), a search and review of INDCs^6^ yields fifteen FFSR references ranging from Burkina Faso to New Zealand (Table 1). Before proceeding, it is helpful to put the references themselves into analytical focus and make sense of what “work” they are doing.

**Table 1:** Countries with INDCs including FFSR references

Region	Countries with FFSR references
East Asia and Pacific	China, New Zealand, Singapore
Middle East and North Africa	Egypt, Morocco, United Arab Emirates, Kuwait, Iran
Sub-Saharan Africa	Burkina Faso, Ethiopia, Ghana, Senegal, Sierra Leone
South and Southeast Asia	India, Vietnam

As described in Table 2, references display remarkable diversity. Some references are clearly being deployed to advertise the ambitiousness of a country’s past efforts on climate: India and Ethiopia highlight successful reform efforts already implemented, Singapore emphasizes the absence of any energy subsidies as a policy success, and New Zealand makes reference to its leadership in the Friends of Fossil Fuel Subsidy Reform.^7^ Other countries make references to substantiate what policies they *would* pursue in a basket of emissions mitigation actions. Where FFSR is more concretely positioned as part of policy planning, the logic of FFSR differs – in cases like Iran, India, and Senegal, reform promises the recovery of fiscal resources that can be re-invested towards climate action. In other instances, including China and Burkina Faso, the purpose of reform is to level the playing field in order to support the scaling of renewable energy. Still, others are offered as an alternative to costly industrial policy when transitioning energy sectors under fiscal constraints. As stated in Burkina Faso’s INDC, their commitment is to “Clearly promote renewable energy, at least by eliminating fossil fuel subsidies and, at best, by subsidising investments in renewable energy” (Burkina Faso, 2015).

**Table 2:** FFSR references in INDCs by section, justification, and commitment

Country	INDC section	Justification for FFSR	Issue specific commitment
Iran	National Contributions—Financial and Technological Needs	FFSR as means of financing GHG mitigation action	None—possible policy option
Kuwait	National Contributions—Laws and Regulations	FFSR as a means of reducing GHG emissions/Qualifying Ambition	None
China	National Contributions—Increasing Financial and Policy Support	FFSR as means of financing GHG mitigation action	Advancing reform in prices and taxation
New Zealand	National Circumstances	Qualifying ambition	None
Singapore	National Circumstances	Qualifying ambition	None
Egypt	Mitigation Policies	FFSR as a means of reducing GHG emissions	As a pillar of future mitigation action
Morocco	National Circumstances/Commitments	FFSR as a means of reducing GHG emissions	Reducing subsidies over and above existing reforms
United Arab Emirates	Economic Diversification with Mitigation Co-Benefits	FFSR as a means of reducing GHG emissions	Adjusting tariffs to reflect cost of generation by 2021
Burkina Faso	National Commitments	FFSR as a means of reducing GHG emissions	As a goal—ambition to eliminate fossil fuel subsidies
Ethiopia	Fairness, Equity, and Ambition	Qualifying ambition	None
Ghana	Mitigation Policy Actions	FFSR as a means of reducing GHG emissions	As a goal—phasing out fossil fuel subsidies
Senegal	Implementation of Mitigation Actions	FFSR as means of financing GHG mitigation action/reducing GHG emissions	None—possible policy option
India	Climate Change Finance Instruments	Qualifying Ambition/FFSR as means of financing GHG mitigation action	None—possible policy option
Sierra Leone	Conditional Contributions	FFSR as a means of reducing GHG emissions	None—possible policy option
Vietnam	Measures to Achieve GHG Mitigation Targets	FFSR as a means of reducing GHG emissions	Implementing roadmap to phase out fossil fuel subsidies

This close examination offers initial support for the analytical purchase of considering NDCs as a tool of credibility as opposed to an individualistic statement of state interests or policy plans. References celebrate its absence, cite leadership on the issue, and use issue references to substantiate the ambition of *prospective* climate policy actions. References rarely tie states to the mast of serious future reform interventions, but rather draw on the appropriateness of the policy idea to justify emission reduction targets without necessarily making new commitments. Further, in line with research on international norms more generally, contextual differences in objectives, institutions of energy sector management, and national circumstances moderate how FFSR is articulated (Acharya, 2004). Differences in how FFSR “fits” resonates with the conception of policy ideas as “symbolic technology” (Laffey & Weldes, 1997); in this case, a variegated tool to signal ambition in ways that are feasible and appropriate given national circumstances and international audiences.

### Context and process: FFSR, the Paris agreement, and INDCs

Moving away from specific references, we look for evidence of broader contextual conditions that may have affected the practical or normative consideration of FFSR as an appropriate policy idea. We identify four major contextual factors: international oil prices, the process of INDC drafting, institutionalization of FFSR and related learning processes, and salience-boosting climate diplomacy.

International oil prices often open and close windows of opportunity for reform (Interview 1, 4, 5). When oil prices decrease, reducing subsidies has a less noticeable impact on final prices for fossil fuel products, and lowers risk of public backlash. During the negotiations of the Paris Agreement, spot prices for oil were relatively low, down from over USD $100 per barrel in 2014 to roughly USD $40 by the end of 2015 (IEA, 2016). Lower oil prices may have softened the perceived political costs of FFSR across the board while INDCs were being drafted.

The INDC process itself was also an important contextual factor. 2015 was characterized by intense negotiations and numerous engagements for country representatives. As one negotiator noted, “it was a bit chaotic” (Interview 6). Countries had a limited window to articulate their INDCs and to finalize the articles of the Paris Agreement itself. For National Contributions, the timelines, frameworks, and commitments were suddenly the remit of states to determine for their own, and capacity for the development of INDCs was variable. Many states called in international organizations and consultants to support the INDC process amid unclear expectations (Interview 1, 2, 4). Though the INDC process was government owned and led, under time and capacity pressures, clear and rigorous technical work from outside sources became an especially valuable resource (Interview 6). Subsidy reform in particular had important issue-linkages for assembling credible plans: reform could convincingly even the playing field for renewable energy deployment commitments and create fiscal space for financing climate action (Interview 15), which we see reflected in the text of references. Fiscal reforms also provided “leverage potential” for lower-income countries seeking further assistance from international financial institutions who considered the issue a priority (Interview 15).

The institutionalization of FFSR as a norm in international climate politics had also advanced significantly by 2015, especially in the context of IOs. In 2009, the G20 Pittsburgh Summit critically galvanized efforts to better understand and address FFSR across a range of organizations, tasking the IEA, the OECD, OPEC, and the World Bank with quantifying and understanding the impact of fossil fuel subsidies.^8^ In the case of the OECD, the commitment mobilized funding to collect data and develop databases, ultimately leading to a prolonged engagement on the issue (Interview 3). Attention built outside of these four organizations as well. For the IMF, though market distorting subsidies had long been an issue of concern, the ascension of Christine Lagarde as Managing Director in 2011 empowered an internal effort to invest more attention to climate-related issues, including fossil fuel subsidies, in lending and macro-economic technical assistance (Skovgaard, 2021, p. 125). The United Nations Environment Programme (UNEP) also took on subsidies, launching green economy assessments for countries that dovetailed with the Rio + 20 UN Conference on Sustainable Development in 2012, which had foregrounded “green economy policies” and “sustainable consumption” (Interview 2). Across international organizations, an increasing recognition of the overlap between fiscal decisions and environmental consequences, and an increasing allocation of staff and resources, focused greater attention on fossil fuel subsidies. By 2015, the notion that fiscal reforms could have environmental co-benefits was increasingly normalized in the international policy community, including among the IOs assisting with INDC drafting—The World Bank, UNEP, and the United Nations Development Programme (UNDP), for instance.

In addition, more successful and durable policy experimentation had taken place between 2010 and 2015 in national contexts and in partnership with international organizations. For IOs, increased issue attention corresponded with clearer lessons about how to address the political dimensions of fossil fuel subsidy removal. Complementary policies demonstrating their effectiveness in reform efforts have been internalized in IO programs and consultations, including widespread public engagement, automatic pricing adjustments, and redistributive compensation to mitigate price shocks (Interview 2, 4, 11, 15). IOs also recognized the importance of engaging stakeholders from across government ministries and even competing political parties, in addition to encouraging policy phase-ins to avoid overloading a country’s “reform stamina” (Interview 15). Some of those IO programs have facilitated peer-learning networks for governments, in part to demonstrate “life after reform” as well as to build country capacity (Interview 4). By 2015, there were more positive lessons to draw from and more available technical assistance for interested countries, to some extent softening the risks otherwise associated with FFSR.

Around the same time, institutionalization in global agenda setting also augmented issue salience. In 2014 and 2015, FFSR became a small but established part of the Sustainable Development Goals (SDGs): “rationalizing” fossil fuel subsidies was an element of both the 2030 Agenda for Sustainable Development (Goal 12.1c on Sustainable Consumption) and the 2015 Addis Ababa Action Agenda for Financing Sustainable Development. The fact that the SDGs and the Paris Agreement were negotiated in rapid succession meant that many of the related outcomes and ideas were close at hand for negotiators and ministers going into COP 21 (Interview 6, 7). It also meant that, as the SDGs assigned particular IOs as custodians for specific indicators, organizations like UNEP (which was assigned to 12.1c) had a mandate to assist countries on that objective across a number of work streams (Interview 2).

FFSR was also made salient in 2015 by state-led climate diplomacy in the form of the Friends of Fossil Fuel Subsidy Reform (FFFSR), “raising the profile of the issue” (Interview 8). New Zealand created the FFFSR in 2010 in response to the G20 commitment, intending to support and hold G20 countries accountable (Rive, 2018). By 2012, membership grew to include Costa Rica, Denmark, Ethiopia, Finland, Norway, Sweden, and Switzerland. In the context of the Paris Agreement, the FFFSR launched a “communiqué” in April of 2015 that articulated the importance of eliminating fossil fuel subsidies as a means of addressing climate change. The specific objective was to accumulate diplomatic clout and to raise awareness in advance of the Paris Agreement’s negotiation (Interview 10). The membership of Costa Rica and Ethiopia, which were also successful experimenters with FFSR, especially helped augment the organization’s influence in the Global South (Interview 6). Members of the FFFSR engaged diplomatically to rally communiqué endorsements, ultimately receiving 27 signatures from countries including the United States, the United Kingdom, and France, among others (FFFSR, 2020). The communiqué effort culminated in several high-profile events: Norway’s Prime Minister Erna Solberg discussing FFSR in a speech during the opening day of COP 21 (Reuters, 2015), a FFFSR-side event during the conference (IISD, 2015), a press-grabbing statement from the Prince of Wales’s Corporate Leaders Group (Corporate Leaders Group, 2015b), as well as formal presenting of the communiqué from then-Prime Minister of New Zealand, John Key, to the Executive Secretary of the UNFCCC, Christina Figueres (Corporate Leaders Group, 2015a). Though these final high-profile events occurred after much of the INDC drafting had been completed, they indicate the degree to which the Friends was able to make FFSR a salient issue.

### Cross-national variation—Country engagement by transnational policy actors

We argue that the increasing institutionalization of FFSR as a norm by 2015 was important, but not an entirely sufficient condition for INDC references, especially given the absence of the issue in the articles of the Paris Agreement itself. Rather, it created the latent possibility that, given the open-ended nature of requirements for INDCs, transnational policy actors could activate the norm by demonstrating how FFSR could be a legitimate and appropriate means of substantiating country commitments. As Risse-Kappen (1994) has convincingly argued, “ideas don’t float freely”, but require actors to make the case for a solution among competing alternatives. To be clear, much of the issue advocacy we document is in the form of technical and economic analysis and not political or moralistic claim-making. Thus, the mechanism of influence is large capacity building and information provision as opposed to mobilizing political pressure, symbolic politics, or naming and shaming (cf. Keck and Sikkink 1998). Still, this technical work had the purpose of supporting the adoption of FFSR as an appropriate means to address climate change.

In this section, we first identify relevant transnational policy actors and evaluate the correspondence between engaged countries and those referencing FFSR in INDCs. In addition, we adjudicate an alternative explanation that would discredit the argument: that norm entrepreneurship, or transnational policy action more generally, is epiphenomenal to political and economic factors conditioning the likelihood that FFSR would be considered. As such, we draw on country-level data from 2015 to evaluate whether political economic factors minimize or moderate empirical patterns we find.

One major transnational policy actor who promoted the norm was the Global Subsidies Initiative and its “Global Subsidies Initiative – Integrated Fiscal” (GSI-IF) Modeling effort, supported by the Nordic Council of Ministers (Merrill et al., 2015). GSI-IF produced economic models for twenty countries on the long-run fiscal and environmental benefits of FFSR (emissions reductions specifically) and delivered findings to country representatives in run-up meetings to COP 21 (Interview 1). Countries were selected for modeling largely based on the proportion of government budgets allocated to subsidies, the size of consumer fossil fuel subsidies, and the emissions reduction potential from removal (Merrill et al., 2015, p. 13).

With the GSI-IF effort, initial work began well in advance in order to have sharable results by the first Subsidiary Body for Scientific and Technical Advice (SBSTA)^9^ meeting in June 2015 (Interview 1). The GSI was present at both SBSTA meetings, June and November, to share its reports with national representatives. Though the scope of this effort was limited by funding and access, GSI was able to engage in discussions with fifteen out of the twenty countries it had modeled, presenting research and results that outlined environmental and economic co-benefits of prospective subsidy reform (Interview 1). GSI representatives identified the importance of providing representatives with research that built on their country’s own emissions profile and energy statistics, as well as presenting results before the deadline to submit INDCs (Interview 1). Undoubtedly, GSI’s history of working and consulting with countries like Vietnam and Morocco prior to Paris, in addition to their reputation as a producer of objective and high-quality research, was an important contributor to their access. Ultimately, representatives from GSI saw evidence of their efforts in the list of referencing countries (Interview 8).

A second major policy actor and source of norm activation was UNEP and its Green Fiscal Policy Network’s “Fiscal Policy Assessments” (specifically in Africa) prior to the Paris Agreement (UNEP, 2020). Building on the momentum of the Arusha Declaration from the African Ministerial Conference on the Environment (IISD, 2012), UNEP conducted case studies and analysis in close partnership with government officials in eight countries between 2010 and 2015 (UNEP 2015). These engagements focused on appropriate actions for climate mitigation and adaptation, including subsidy reform, and continued in the lead up to COP 21 (Interview 2). Though UNEP Fiscal Policy Assessments went beyond targeting African countries in that period, assessment reports and engagement in countries like Serbia or Barbados did not reference fossil fuel subsidies as part of policy analysis or planning, based on a survey of publicly available documents.

We also find evidence of UNEP’s impact. Though UNEP did not advocate for FFSR in the COP 21 negotiations directly, the technical assistance and macro-economic research produced on green economy reforms between 2011 and 2015 were readily available, long-term in nature, and something countries could get further technical assistance with. Key program coordinators saw evidence of their work getting taken up in national policy plans on sustainable development (Interview 2), which were often the cited groundwork for INDC mitigation actions (e.g., Ghana’s “Shared Growth and Development Agenda”, Egypt’s “Green Egypt Vision 2030”).

Table 3 illustrates the “success rate” of countries engaged by either GSI-IF or the UNEP programming and FFSR references (see Appendix 2 for country lists). Given that only 15 out of 192 Paris signatory countries included subsidy reform references, referencing countries are especially well represented in association with these two sources of norm promotion. Dichotomous variables, even with low event counts, also lend themselves to statistical tests of association: using likelihood ratio tests for small event counts, being a recipient of the GSI-IF economic modeling effort and INDC FFSR inclusion is associated and statistically significant at the 0.05 level ($\chi^{2}$= 20.06, *p* < 0.001), as is the association with UNEP’s Green Assessment Program ($\chi^{2}$= 11.00, *p* < 0.001).

**Table 3:** Sources of country engagement on FFSR and “success rate” for FFSR INDC references

Engagement source	“Success” rate
GSI-IF	8/20–40%
UNEP programming	4/8–50%

Other international organizations and initiatives were considered as FFSR advocates but excluded for conceptual and empirical reasons.^10^ While they contributed to the broader institutionalization of the norm—and thus were part of the normative environment from which references were drawn—they were not instrumental as policy actors activating state interest in the INDC process. The OECD and IEA have focused on FFSR, but largely through hands-off data collection and analysis (Interview 3). The World Bank’s Energy Sector Management Assistance Program (ESMAP) provides technical assistance to countries considering reform but reportedly did not advocate for particular policy solutions in the INDC/NDC process (Interview 4). Indeed, the countries in ESMAP’s Energy Sector Reform Facility program prior to 2016 did not correspond to countries with FFSR INDC references (Likelihood Ratio Test, $\chi^{2}$ = 0.218, *p* = 0.641). As a critical catalyst for fiscal reforms, the IMF also plays an important role, and it advocates for policy solutions in regular Article IV reviews.^11^ Though FFSR is largely framed as a fiscal policy issue and not an environmental one in these consultations, as a check, 2015 consultation reports were reviewed for FFSR-related policy recommendations. Though 37 out of 114 country consultations included recommendations relating to energy or fossil fuel subsidy reform, countries who received FFSR-related policy recommendations in their IMF consultations were not more likely to reference FFSR in their INDCs (Likelihood Ratio Test, $\chi^{2}$ = 1.022, *p* = 0.311). Socialization in organizations where FFSR was an agenda item was also considered. This included the G20 and APEC with their peer review process for rationalizing fossil fuel subsidies, membership in the Friends of Fossil Fuel Subsidy Reform,^12^ and signatories of the FFFSR communiqué. Though arguably none of these groups engage in any kind of country-targeted policy advocacy, there was some overlap between these groups and INDC references. However, likelihood ratio tests suggest that in each instance, their correspondence is not significant at a 0.05 significance level.

It is important to note that the sources of issue advocacy identified do not offer a *deterministic causal account* of policy transfer. Some engagements overlapped, some attempts at issue advocacy failed; countries have agency and interests all the same. Nonetheless, the results do suggest that targeted policy advocacy increased the likelihood of FFSR referencing, built on a growing norm, and demonstrated its use in bolstering the credibility of GHG emissions reduction commitments. Given that engaged countries were not chosen because of pre-disposition towards reform but rather the quantifications of consumer subsidies or the country’s economic importance, we should take seriously the correspondences we observe.

However, it is worth asking if correlations between transnational advocacy and FFSR references are epiphenomenal to, or modified by, political economic factors reviewed in Sect. 2. Though evaluating this question in a multivariate regression framework with such low event counts is fraught, we can examine differences and similarities between countries based on relevant variables. First, we compare countries engaged by transnational policy actors and countries that were not engaged but have similar economic and institutional profiles, examining differences in the rate of INDC FFSR referencing. If advocacy by transnational policy actors is epiphenomenal, then we would expect to see similar rates of referencing between the engaged and un-engaged across comparison groups. Second, we consider political economic factors as a potential moderator by comparing countries who were engaged by transnational policy actors and made references to engaged countries who did not. If norm entrepreneurship is moderated by country characteristics, we should observe sizeable differences in measures of central tendency.

In both analytical steps, we evaluate country differences on six dimensions: authoritarianism/democracy as measured by the Economist Intelligence Unit’s Democracy Index, indebtedness measured by debt to GDP ratios from IMF data, national income as measured by GDP per capita from World Bank data, the size of incumbent domestic fossil fuel industries as measured by the U.S. Energy Information Administration dataset on global petroleum production in millions of barrels/day (normalized to GDP), existing subsidies as measured by pre-tax consumer petroleum subsidies as a percentage of GDP calculated by the IEA, and fuel import dependence as the difference between petroleum production and consumption in millions of barrels/day (IMF, 2020; International Energy Agency, 2021; Kaufman & Kraay, 2020; The World Bank, 2020; U.S. Energy Information Administration, 2020).

To analyze the effect of issue advocacy net of political economic factors, a nearest-neighbor matching algorithm^13^ was used to construct a “treatment” group (23 countries engaged by either GSI-IF and/or UNEP^14^) and a “control” group (a selection of two control cases per treated country that most closely match on the six political and economic variables) (See Appendix 3 for information on the balance of the matched data). In the resulting sample of 69 countries, the proportion of INDC FFSR references in the treatment group is 43% (10/23), while the proportion of references in the comparison group is 4.5% (2/46); a statistically significant difference (Likelihood Ratio Test, $\chi^{2}$ = 15.81, *p* < 0.001). Though the sample balance is not entirely even, for countries with similar characteristics, issue advocacy is much more likely to correspond to INDC FFSR references.

**Fig. 1:**
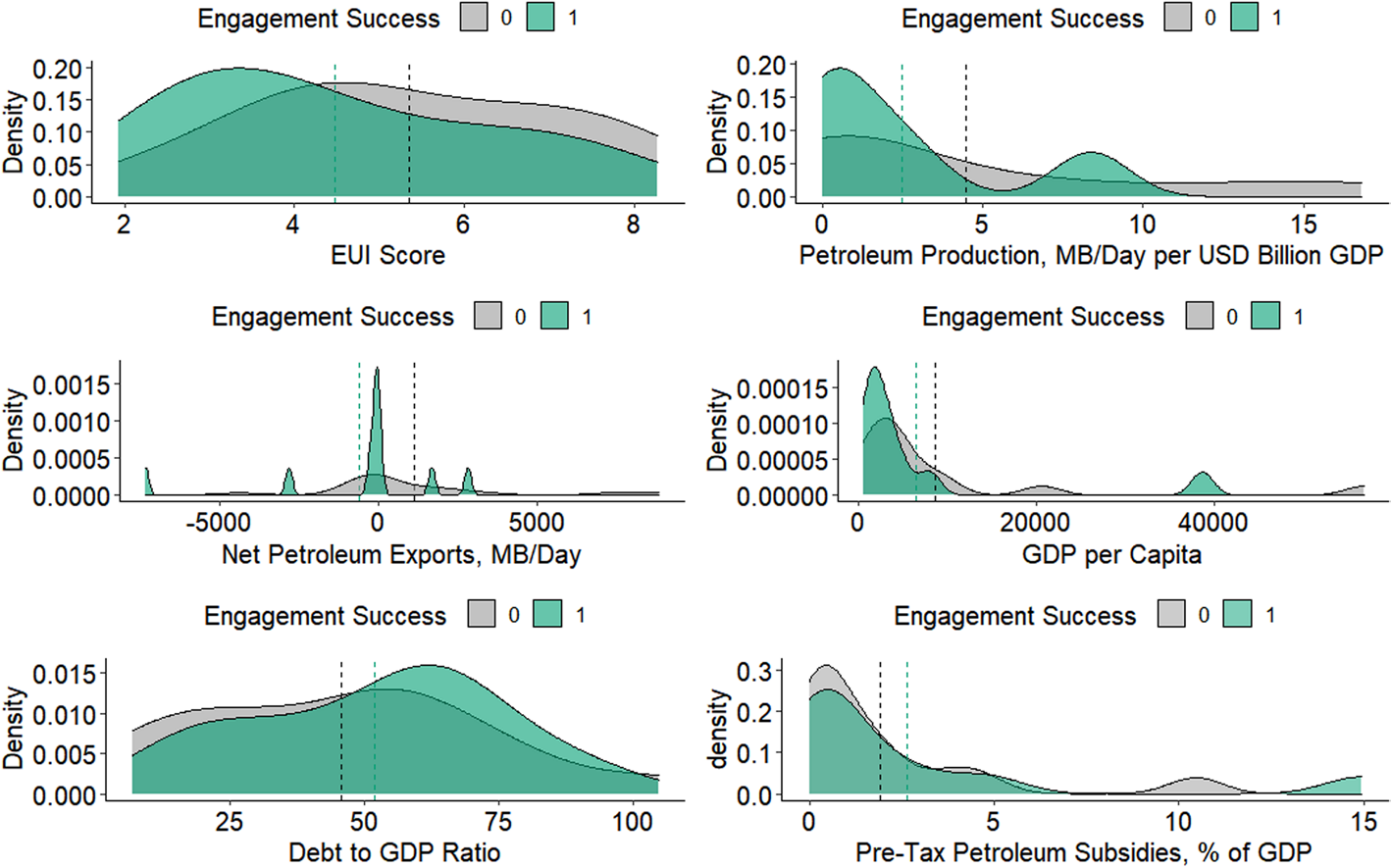
Density plots and mean values for key economic and political variables, engagement “Successes” and “Failures”

As for the possibility of political economic moderation, we consider whether countries that are engagement “successes” (engagement by norm supporting policy actors and FFSR referencing, *n* = 10) differ from countries that are engagement “failures” (engagement by norm supporting policy actors but no FFSR referencing, *n* = 13). Mean values on political and economic characteristics are visualized with density plots in Fig. 1, with corresponding dotted vertical lines to indicate mean group values. Political economic priors that we discuss in Sect. 2 are directionally correspondent on several dimensions: countries that were engaged by norm-supporting transnational policy actors but did not reference FFSR in their INDCs produced more petroleum on average, had lower debt-to-GDP ratios, and were generally net petroleum exporters. On the other hand, engaged countries that referenced FFSR were also poorer, less democratic, and had higher petroleum subsidies, conflicting with some findings from the political economy literature. Though the differences are suggestive, Wilcoxon Rank Sum Exact tests^15^ show that none of the variation between groups on the six variables examined is statistically significant at the 0.05 level (Appendix 4). Overall, this suggests that there may have been some weak moderation by political and economic country characteristics, but it is not clear that there is any systematic effect.

In sum, we find that countries targeted by transnational policy actors were more likely to reference FFSR. ﻿﻿﻿Interestingly﻿, activation of norms followed different pathways and sequences: with the GSI, the case was made for the emissions reduction potential of FFSR during the drafting of INDCs. ﻿With UNEP, a more prolonged engagement and integration of green fiscal policies in national policy plans enabled activation (or re-activation) of the norm in the context of the Paris Agreemen﻿t. These correspondences were resilient even when comparing samples of countries with similar institutional and economic characteristics. When parsing advocacy “successes” and “failures”, we also do not find evidence of strong moderating effects from variables like net petroleum exports or GDP. Given the texts of references and the institutionalization of the norm in international politics, this lends further credence to our overall argument.

## Discussion and conclusions

This paper addresses the question of how a diverse group of fifteen countries elected to include references to a thorny issue like FFSR in their INDCs. We argue that the Article 4 process generates a credibility dilemma: states need to put forward ambitious mitigation commitments but also need to find appropriate policies and positions that can substantiate their emissions reduction plans. In response, we explain FFSR references in two steps: first, states will refer to actions insomuch as those actions have been legitimated and institutionalized internationally. Second, increasing institutionalization can open a window of opportunity for norm activation as transnational policy actors articulate how related policies can appropriately justify commitments. In large part, we find evidence to support this argument. The texts of INDCs reveal that FFSR references qualify national ambition or are offered as credible policy options to achieve mitigation targets, though substantial reform commitments are scarce. We identify convergent contextual factors making FFSR appropriate and salient including the institutionalization of FFSR as a norm in international environmental agreements, initiatives, and programs of various international institutions; as well as salience-boosting climate diplomacy. Economic conditions (oil prices) and bureaucratic constraints in the INDC process also appeared relevant for softening the perceived risks of FFSR and augmenting the demand for external technical assistance, respectively. Finally, referencing countries are disproportionately represented among states engaged by relevant FFSR issue advocates regardless of cross-national differences in political and institutional characteristics. Such characteristics also do not systematically moderate the “success” of norm activation. In sum, norm institutionalization and issue advocacy work in conjunction to explain what we observe.

One might ask why we don’t observe *more* references across countries. Countries are likely to be risk-adverse about what they are willing to commit to in international fora: even in INDC references in our study, stringent commitments are few and far between, suggesting efforts to draw on the credibility of policy ideas while also hedging against issue-specific targets that might be politically difficult to actualize. Further, to the extent that issue advocacy is catalytic, it is limited by the resources available to the issue-specific programs and organizations like GSI. Another possibility is that using price liberalization as a means of achieving climate targets is a relatively new policy frame. Paris was only six years on from the 2009 G20 Pittsburgh Declaration, the year in which the GSI also shifted focus from biofuel subsidies to fossil fuel subsidies (Lemphers et al., 2018). The climate linkage may not have been apparent for governments less closely integrated into networks where the issue was considered important for environmental (as well as fiscal) reasons. To the extent that the need to signal ambition on an issue is contingent on the relationship between states and a critical international audience, countries who recognize the linkage may nevertheless be unconcerned: the ability of G20 countries to finance infrastructure is less contingent on their standing with the IMF, for instance. Whatever the causes, further normalization is likely needed to overcome existing constraints. For that reason, it was significant that at COP 26, FFSR entered into the intergovernmental texts of the UNFCCC for the first time in the Glasgow Climate Pact, calling on countries to phase out inefficient fossil fuel subsidies (UNFCCC, 2021). As Harro van Asselt argues (2021), this development is “breaking a taboo”, demonstrating a recent and hard-fought consensus on FFSR as a “credible climate change mitigation measure”. Given the framework we articulate, this should make activation easier and commitments more widespread moving forward.

Several limitations and extensions are worth noting, especially for future research. First, our analysis pays attention to the normative conditions that could explain referencing FFSR in INDCs. It does not reconstruct the decision-making process of countries nor does it “get in the heads” of ministers drafting their Contributions. This is, nonetheless, important work: recent research efforts have examined the inside track of NDCs and found, for instance, that Argentina’s second NDC was reportedly motivated by a desire to improve its international reputation and The Gambia’s to “make a mark” on climate negotiations (Dash & Gim, 2019). In our case, it limits our ability to draw conclusions about how norms are exerting themselves exactly: for instance, whether norms matter because countries are persuaded that reform is an appropriate means of decarbonization, or whether there might be some strategic opportunism for, say, opening the door to greater concessional financing from IOs that prioritize the issue. Such steps are important for reconciling remaining puzzles about why issue advocacy works in some cases and not others or to identify pathways of influence outside the scope of our study. Additional granularity is also needed in disaggregating fuel types among subsidies: though we deal with FFSR in broad strokes, subsidies for coal versus petroleum, for instance, may have distinct political dynamics when it comes to international environmental agreements and the Paris Agreement specifically. Such disaggregation is not only important for research, but for policy planning as well (van Asselt & Skovgaard, 2021).

Finally, these results join an increasing chorus of scholarship calling into question oft-cited rational-institutionalist conceptualizations of the Paris Agreement (see Keohane & Victor, 2016). If NDCs are the consequence of rushed negotiators or consultations with IOs, the extent to which the results represent the atomistic interests of the state, as opposed to something more relational and co-constitutive, is an open and empirical question. Understanding the credibility dilemma and the force of norms to guide the content of NDCs also helps reconcile justifiable skepticism about the possibility of over-inflated commitments (e.g., Bang, Hovi, & Skodvin, 2016)—states may pronounce ambitious targets, but substantiate their plans with salient policy ideas that they may have difficulty implementing. This is an important reflection of international environmental agreements more generally, and deserves ongoing monitoring and investigation, especially if the Article 4 approach of the Paris Agreement becomes a template for future treaties and regimes.

## Appendix 1

Interviews

- Interview 1—Former IISD Employee. August 27th, 2020.
- Interview 2—Economist, UNEP. September 11th, 2020.
- Interview 3—Ronald Steenblik, Senior Fellow, IISD and Former Special Councilor for Fossil Fuel Subsidy Reform, OECD. August 21st, 2020.
- Interview 4—Economist, The World Bank. August 4th, 2020.
- Interview 5—Philip Gass, Lead, Transitions, IISD Energy Program. August 8th, 2020.
- Interview 6—Consultant, Ministry of Environment and Energy, Costa Rica. September 1st, 2020.
- Interview 7—Consultant, Ministry of Environment and Energy, Costa Rica. September 1st, 2020.
- Interview 8—IISD Employee. July 12th, 2020.
- Interview 9—Economist, the International Monetary Fund. October 1st, 2020.
- Interview 10—Malena Sell, Ministry of Foreign Affairs, Finland. October 21st, 2020.
- Interview 11—Analyst, Indian Environmental NGO. October 26th, 2020.
- Interview 12—Eike Meyer, Advisor, GIZ. November 6th, 2020.
- Interview 13—Program Manager, Canadian Environmental NGO. November 6th, 2020.
- Interview 14—Analyst, Indian Environmental NGO. November 22nd, 2021.
- Interview 15—Advisor, UNDP. December 14th, 2021.

## Appendix 2

GSI-IF and UNEP engagements

**Table:**

Program	Countries
GSI-IF	Algeria
	Bangladesh
	China
	Egypt
	Ghana
	India
	Indonesia
	Iran
	Iraq
	Morocco
	Nigeria
	Pakistan
	Russian Federation
	Saudi Arabia
	Sri Lanka
	Tunisia
	United Arab Emirates
	United States
	Venezuela
	Vietnam
UNEP Fiscal Policy Program (Africa)	Burkina Faso
	Egypt
	Ghana
	Kenya
	Mauritius
	Rwanda
	Senegal
	South Africa

## Appendix 3

Nearest neighbor matching and sample balance

**Table:**

Variable	Mean, population	Mean, treated	Mean, control	Std. mean difference	Variance ratio	eCDF mean	eCDF max
Democracy score	5.3334	4.8957	5.362	− 0.2479	0.9645	0.0579	0.2174
Petroleum production, millions of barrels/day per USD billion GDP	1.8407	3.2942	2.3127	0.2027	1.3739	0.0896	0.2174
Net petroleum exports, millions of barrels/Day	17.4305	452.4888	95.6136	0.1089	13.8121	0.1215	0.3043
GDP per capita, USD	14,498.78	8002.886	11,658.25	− 0.2708	0.614	0.0844	0.2609
Debt-to-GDP ratio	54.9198	46.8353	49.4802	− 0.0973	1.7471	0.0784	0.2609
Pre-tax petroleum subsidies, % of GDP	1.3427	1.8683	1.3464	0.1631	1.5674	0.1295	0.3261

## Appendix 4

Wilcoxon rank sum exact tests

**Table:**

Variable distributions tested (Engagement and FFSR reference vs. Engagement and no FFSR reference)	Test statistic	*P*-Value
Democracy score	59	0.2857
Petroleum production, millions of barrels/Day per USD billion GDP	60.5	0.5981
Net petroleum exports, millions of barrels/Day	67	0.5169
GDP per capita, USD	59	0.2857
Debt-to-GDP ratio	94	0.4838
Pre-tax petroleum subsidies, % of GDP	69	0.9766

## Footnotes

1 In this paper we refer to NDCs as a description of the broader category of submissions over time, and INDCs as the specific initial submission in 2015/2016.

2 The GSI is a think tank that conducts research and engages in consultations on fossil fuel subsidies and their reform, launched by the International Institute for Sustainable Development (IISD). https://www.iisd.org/gsi/.

3 Ratification of the Paris Agreement converted INDCs to the status of NDCs. Recall that we use the term NDC to describe the category of national submissions over time, in contrast to INDCs which specify the first instantiation of submissions in 2015/2016 pre-ratification.

4 Article 2a of the Paris Agreement states the objective is to “hold the increase in the global average temperature to well below 2 °C above pre-industrial levels” (UNFCCC, 2015, p. 3).

5 Our use of the term “norm activation” diverges from, but has similarities to, its use in social psychology. There, the norm activation model refers to predicting individual pro-social behavior on the basis of personal norms, awareness of consequences, and feelings of responsibility (Van Der Werff & Steg, 2015).

6 We use INDCs because all countries submitted in roughly the same period, allowing for more precision about pathways of influence. NDCs, conversely, were submitted across 2016–2020 as countries ratified the Paris Agreement domestically, in some cases making amendments. Overall, Merill et al. review FFSR-related changes from INDCs onward and finds that the references are “largely the same countries as in 2015 with some minor changes” (2019, p. 22).

7 The Friends of Fossil Fuel Subsidy Reform is a group of states who advocate through international diplomacy. They are discussed in more detail in the subsequent section.

8 For a discussion of why G20 commitments haven’t been more catalytic for member countries themselves, see Skovgaard (2021).

9 The SBSTA provides expert consultations to countries on matters relating to agreements under the UNFCCC.

10 We acknowledge that diverse sources of targeted norm entrepreneurship or organizational membership could operate interactively. Exploring whether that is the case is outside the scope of this paper but is worth exploring in future research.

11 Article IV reviews are bilateral consultations with member countries on economic policies and development, often held annually.

12 However, New Zealand’s leadership of the FFFSR clearly explains their reference, given the text of their INDC.

13 Nearest neighbor matching is implemented with the Mahalanobis distance, as opposed to a propensity score estimated by logistic regression.

14 There are 26 countries in total that were engaged by either GSI or UNEP, as described. 3 “treated” countries were omitted due to missing data in the covariates of interest.

15 A non-parametric test for differences in distributions with small sample sizes.
